# Industrial Thermal Insulation Properties above Sintering Temperatures

**DOI:** 10.3390/ma14164721

**Published:** 2021-08-21

**Authors:** Amalie Gunnarshaug, Maria-Monika Metallinou, Torgrim Log

**Affiliations:** 1 Q Rådgivning AS, Øvregata 126, 5527 Haugesund, Norway; amg@q-rad.no; 2 Department of Physics and Technology, University of Bergen, 5020 Bergen, Norway; 3 Fire Disasters Research Group, Department of Safety, Chemistry and Biomedical Laboratory Sciences, Western Norway University of Applied Sciences, 5528 Haugesund, Norway; torgrim.log@hvl.no; 4 Gassco Inc., Bygnesvegen 75, 4250 Kopervik, Norway

**Keywords:** fire testing, heat transfer, thermal insulation, thermal conductivity, transient plane source method

## Abstract

Processing highly flammable products, the oil and gas (O&G) industry can experience major explosions and fires, which may expose pressurized equipment to high thermal loads. In 2020, oil fires occurred at two Norwegian O&G processing plants. To reduce the escalation risk, passive fire protection may serve as a consequence-reducing barrier. For heat or cold conservation, equipment and piping often require thermal insulation, which may offer some fire protection. In the present study, a representative thermal insulation (certified up to 700 °C) was examined with respect to dimensional changes and thermal transport properties after heat treatment to temperatures in the range of 700 °C to 1200 °C. Post heat treatment, the thermal conductivity of each test specimen was recorded at ambient temperature and up to 700 °C, which was the upper limit for the applied measurement method. Based on thermal transport theory for porous and/or amorphous materials, the thermal conductivity at the heat treatment temperature above 700 °C was estimated by extrapolation. The dimensional changes due to, e.g., sintering, were also analyzed. Empirical equations describing the thermal conductivity, the dimensional changes and possible crack formation were developed. It should be noted that the thermal insulation degradation, especially at temperatures approaching 1200 °C, is massive. Thus, future numerical modeling may be difficult above 1150 °C, due to abrupt changes in properties as well as crack development and crack tortuosity. However, if the thermal insulation is protected by a thin layer of more robust material, e.g., passive fire protection to keep the thermal insulation at temperatures below 1100 °C, future modeling seems promising.

## 1. Introduction

The process industry may represent a major accident hazard, e.g., an ignited hydrocarbon leak, resulting in an explosion, or a jet fire exposing adjacent equipment for a prolonged time period. In 2020, two fires occurred in the Norwegian process industry, i.e., on September 28th at the Hammerfest LNG plant [1,2] and on December 2nd at the Tjeldbergodden methanol plant [3,4]. In order to inhibit further escalation, critical equipment is often protected with a layer of passive fire protection. This is sometimes applied in addition to thermal insulation for heat or cold conservation required to maintain the optimal operating temperature [5,6,7]. This thermal insulation may indeed provide some reduction in heat flux to fire-exposed pipes and equipment.

The application of passive fire protection may vary between different countries and companies. A main concern with using passive fire protection is corrosion under insulation (CUI), if not designed and maintained correctly [8]. Excessive fire protection should be avoided by applying fire protection only where strictly required [9]. Hence, there are several factors that should be evaluated before applying the PFP. The international recommended practice for application of passive fire protection is given by API RP 2218 [10], where the applied material, i.e., fire-resistive barrier systems for electrical system components, shall be certified according to ASTM E1725-19 [11]. It runs either according to the E119 or the E1529 temperature curves. The first of these, which does not reach 1000 °C within 1 h, corresponds to the temperatures of representative building fires, while the second curve reaches a constant temperature of 1100 °C after about 5 min, which is typical for hydrocarbon pool fires. These are fires of lower severity than the high-intensity fires addressed in the present study where temperatures of 1200 °C may be expected over prolonged periods.

There are several types of passive fire protection materials on the market, depending on the equipment to be protected. For fire protection of structures in the O&G industry, materials such as Chartek or Fendolite are widely used. The lightweight Fendolite is usually applied as a cementitious spray to structural elements. The epoxy-based Chartek is also mainly sprayed onto these elements for structural fire protection. In some cases, Chartek was previously used also for the fire protection of process equipment. For the protection of pipes or vessels, mineral-based passive fire protection is most often used.

In the oil and gas industry, an unacceptable rupture is often defined as a rupture that may cause fatalities or serious injuries, a rupture that may hinder escape or evacuation, or a rupture that may lead to major additional economic losses [12]. An effective blowdown system is the preferred solution to prevent escalation rather than using passive fire protection [13]. However, in some cases, the blowdown time is too long to avoid a rupture before the system is sufficiently depressurized. In those situations, passive fire protection is required. The general acceptance criteria for the passive fire protection are to avoid rupture of the protected equipment, until the system is depressurized and/or personnel have been evacuated [5]. For simplicity, rupturing or severe structural damage are typically assumed to take place at temperatures above 400 °C, i.e., where the steel types in use start to lose much tensile strength with further temperature increase.

Thermal insulation is widely used in several industries, especially the building industry. There are several studies investigating the thermal properties of thermal insulation [14,15,16,17]. Recently, new materials such as silica-based aerogel were also tested and implemented for combined heat insulation and fire protection of buildings [18]. These were, however, within the operating range of the industrial thermal insulation of interest, i.e., at temperatures below 700 °C, or within temperatures and heat fluxes associated with building fires.

**Figure 1:**
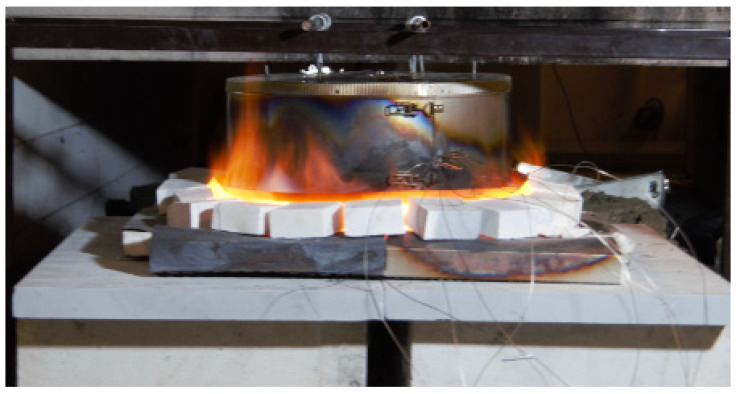
Small-scale fire test set-up as suggested in [19,20].

**Figure 2:**
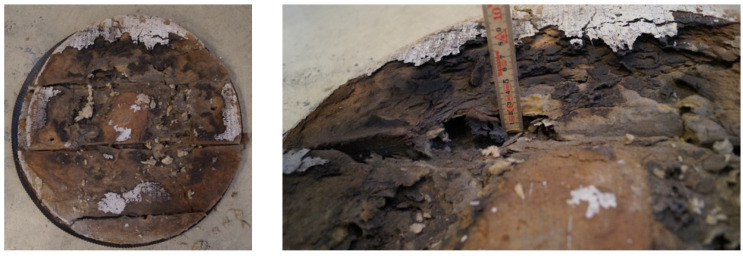
Thermal insulation after jet fire exposure as suggested in [19,20].

Only a few studies involve industrial thermal insulation for the O&G industry at temperatures relevant for high-intensity fires [18,19,20,21,22]. Previous small-scale jet fire tests [19,20] demonstrated that industrial thermal insulation alone, at least for some limited period, may provide sufficient protection against high-intensity hydrocarbon fires. Though degrading significantly at temperatures above 1100 °C, a 50 mm layer of industrial thermal insulation alone, protecting a 16 mm steel wall, may provide sufficient fire protection for a period of 20 min [19,20]. The small-scale test set-up giving cladding temperatures of 1200+ °C is shown in Figure 1. After heat exposure, the thermal insulation had sintered and partly melted, resulting in cracks (see Figure 2).

Fire testing is time consuming and expensive. Several numerical models for the calculation of fire resistance have therefore been provided, typically for walls or steel columns [23,24,25]. For estimating the performance of industrial thermal insulation in high-intensity fire scenarios, a numerical model with input based on the heat treatment of the thermal insulation may be very beneficial. Such a model could then be used to evaluate whether thermal insulation alone would provide a sufficient delay in equipment or piping overheating versus given temperature acceptance criteria in real blowdown scenarios. It could also be used to investigate creative solutions to make the system more robust to fire and, thus, prolong the time needed for depressurizing exposed hydrocarbon-containing equipment to prevent unacceptable ruptures. This is of special interest in aging sites which may not have been designed to be sufficiently robust, and where, e.g., upgrading blowdown and flare systems is associated with a very high cost. To prepare for future numerical modeling, information about changes in thermal conductivity during heating, as well as dimensional changes, of the thermal insulation needs to be known.

In the present study, investigations on the industrial thermal insulation have therefore been undertaken to develop necessary data for future numerical modeling. This includes analysis of thermal transport properties and dimensional changes for test specimens heat treated up to 1200 °C, i.e., at temperatures representative for high-intensity O&G fires. In Section 2, the materials and methods are presented. The results are presented in Section 3 and discussed in Section 4, along with possible future initiatives.

## 2. Materials and Methods

### 2.1. The Thermal Insulation Studied

**Figure 3:**
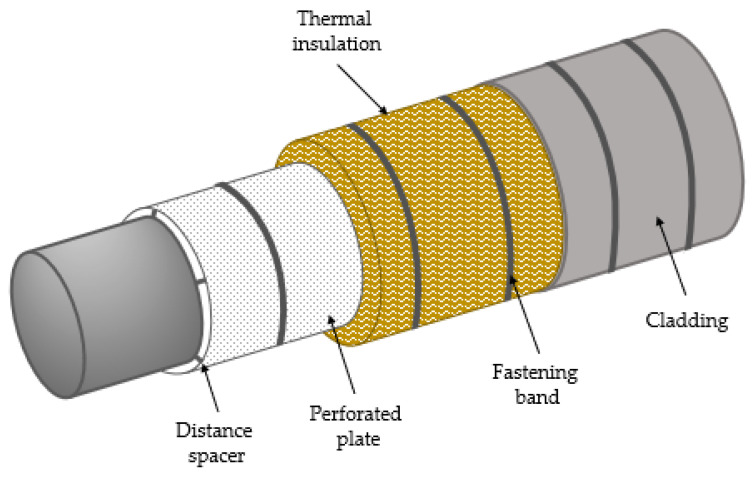
Principle sketch of thermal insulation methodology for corrosion prevention according to [26].

Previously, the thermal insulation was applied directly to steel pipes and equipment. These pipes and equipment were protected from corrosion by paint. It was, however, discovered that when the corrosion-protective paint was exposed to wet thermal insulation over long periods, it lost its corrosion-protective function. Over time, this thermal insulation method therefore resulted in corrosion attacks especially when soaking wet thermal insulation was in contact with pipework and equipment. Thus, the current way of applying thermal insulation allows for an air gap between the object to be protected and the thermal insulation, thus preventing direct contact with potentially soaked thermal insulation (see Figure 3).

Distance spacers, of, e.g., polytetrafluoroethylene are used to create a 25 mm (one inch) air gap between the pipe (or equipment) and the thermal insulation. As seen in Figure 3, a perforated stainless-steel plate supports the thermal insulation to maintain the 25 mm air gap. The system is covered by watertight weather protection, which in industries processing combustible media usually consists of a 0.7 mm-thick stainless-steel cladding.

In the present work, the thermal insulation studied was 50 mm Rockwool (ProRox PSM 971, 50 mm, Rockwool, Hedehusene, Denmark. This product is extensively used in the Norwegian O&G industry for heat or cold conservation. It has also been examined in other recent studies [19,20,21,22]. The detailed technical data and thermal conductivity of the studied thermal insulation up to 350 °C are presented in Appendix A, Table A1 and Table A2. The insulation has, according to manufacturer data, a maximum operating temperature of 700 °C. The focus of the present study was therefore at temperatures above the 700 °C maximum service temperature, and up to 1200 °C, i.e., temperatures associated with high-intensity fires in the O&G industry.

The thermal insulation consists mainly of inorganic oxides, where silica, alumina, magnesia, calcium oxide and iron (III) oxide represent the main components. In addition, there are minor amounts of sodium oxide, potassium oxide, titanium oxide and phosphorous pentoxide. The detailed chemical composition is presented in Appendix A, Table A3.

The production of the thermal insulation involves melting the raw materials at 1500 °C before it is spun, cooled to threads, and woven into insulation mats [27]. Bakelite, i.e., polyoxybenzylmethylenglycolanhydride (C_6_H_6_O · CH_2_O)_x_, is added to give some strength to the thermal insulation at temperatures below the maximum service temperature. To make the material easier to handle, a dust binder (mineral-based oil) is also added.

Upon heating, the dust binder will gradually pyrolyze/evaporate. Bakelite is a plastic material formed through the reaction of phenol with formaldehyde, followed by cross-linking of the polymeric chains. The number of crosslinks (easily affected by small anomalies in the production process) and the presence of other components mixed into the resin affect its degradation process and temperature [28]. In general, a non-balanced reaction may express the degradation of Bakelite:

(C_6_H_6_ ∙ CH_2_O)_n_ **→** CO_2_ + CO + H_2_O + C_soot_ + other products, (1)

Generally, mineral-based thermal insulation has a high porosity, defined by the spaces between the individually woven fibers consisting of a mix of the previously mentioned inorganic salts. Given a nominal thermal insulation density of 140 kg/m^3^, and inorganic salts with densities about 20 times this value, the porosity fraction is about 95% of the volume. This high porosity results in a very low ambient-temperature thermal conductivity of the thermal insulation mats.

The thermal conductivity of highly porous materials at ambient temperature is largely limited by heat transfer through the pores, which are normally too small to exhibit significant within-pore-convection. However, as the temperature increases, the radiation through the pores may start to dominate the local pore heat transfer, which would then, by theory for small pores, increase with a factor *T*^3^, where *T* is the absolute temperature (K) [29,30]. In a previous study [22], it was shown that the thermal conductivity of the insulation studied in the present work could indeed be expressed by the simple relationship *a* + *b*∙*T*^3^.

With increasing temperature, the thermal conductivity of crystalline materials generally passes through a peak in thermal conductivity and then experiences a decay with increasing temperatures due to the mean free path limitation of the phonon interactions. Thus, above the maximum, the thermal conductivity decays as 1/*T* (K^−1^) for increasing temperatures [29]. For amorphous materials, the thermal conductivity is in general much lower, and increases quite linearly with temperature [29]. It may therefore be assumed that the thermal conductivity of the amorphous inorganic salt fibers exhibits a linear function of temperature, i.e., increases modestly with increasing temperature.

At temperatures above 700 °C, the thermal insulation is known to start sintering, and when approaching the eutectic temperature of the salt mixture, it will gradually start melting. The pores gradually collapse with increasing temperatures, making this a very complex system. It should also be noted that it passes through the glass transition temperature somewhere in the range 850 °C to 900 °C. Such a complex system is best analyzed experimentally to understand the involved thermal insulation degradation mechanisms.

### 2.2. Heat Treatment of Thermal Insulation Test Specimens

To investigate the dimensional changes and the breakdown temperature of the thermal insulation when exposed to temperatures representing a high-intensity fire, as in [19,20], muffle furnace tests were conducted based on previous successful studies [22].

In order to avoid any issues with elasticity, 50 mm × 50 mm × 50 mm cubic test specimens were pre-cut two days prior to the heat treatment in a muffle furnace (Laboratory Chamber Furnace, Thermconcept GmbH, Bremen, Germany). The highest temperature of interest in the present study was 1200 °C, i.e., well within the maximum temperature range of the furnace (1300 °C). Just prior to the heat treatment, the height and width of all four sides of the cubic test specimen were measured and noted as references for possible dimensional changes after the heat treatment.

It should be noted that for the heat treatment up to 1200 °C, a test specimen size of 75 mm × 75 mm × 50 mm (height) was used due to the massive loss in height and width at this temperature. This large size was required to perform thermal conductivity measurements of this sample.

**Figure 4:**
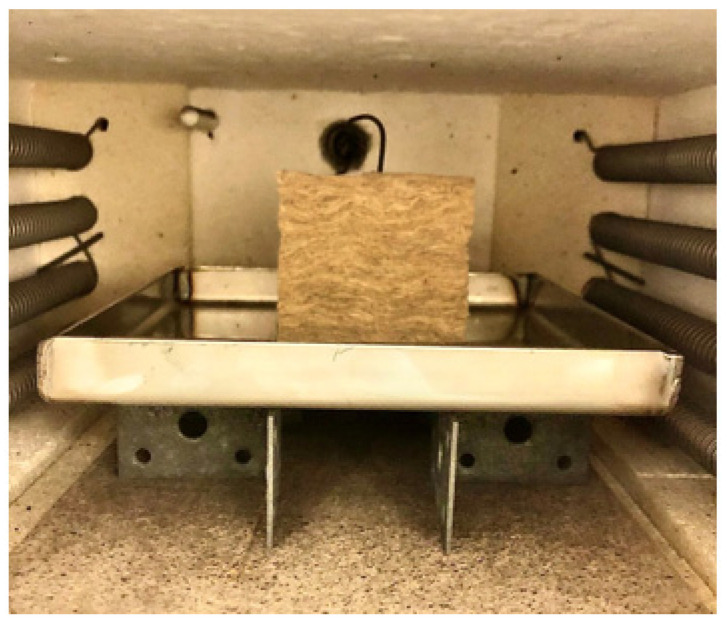
Muffle furnace test setup.

Two thermocouples (type K, mantel, 1.5 mm diameter, Pentronic AB, Västervik, Sweden) were used during the heat treatment. One was placed vertically into the center of the thermal insulation test specimen, and the other one recorded the furnace air temperature. The insulation test specimen was placed on a steel plate, lifted approximately 35 mm above the 15 mm bottom plate as shown in Figure 4, allowing uniform test specimen heating.

A heating rate of 15 K/min was applied to heat the test specimens from ambient temperature to respective maximum holding temperatures in the range of 700 °C to 1200 °C. The holding time at the maximum temperature was 30 min. After heat treatment and cooling of the oven to below 100 °C, the dimensions of the four vertical cube surfaces were again measured at three locations, both in width and height. The average width and height were reported for each test specimen. It should be noted that tests were also performed with vertically aligned test specimens (referring to the thermal insulation mat), but the main focus was kept on tests resembling the fire test set-up shown in Figure 1.

### 2.3. Thermal Conductivity Measurements

The virgin industrial thermal insulation is a pours material, with low thermal conductivity at ambient temperature. Since the pore radiation dominates the internal pore heat transfer, the thermal conductivity is a function of absolute temperature to the third power [28]. For the tested thermal insulation, the conductivity is given by:

*k*_iso_ = 0.034 + 0.311∙10^−9^∙*T*^3^ (W/mK), (2)

**Figure 5:**
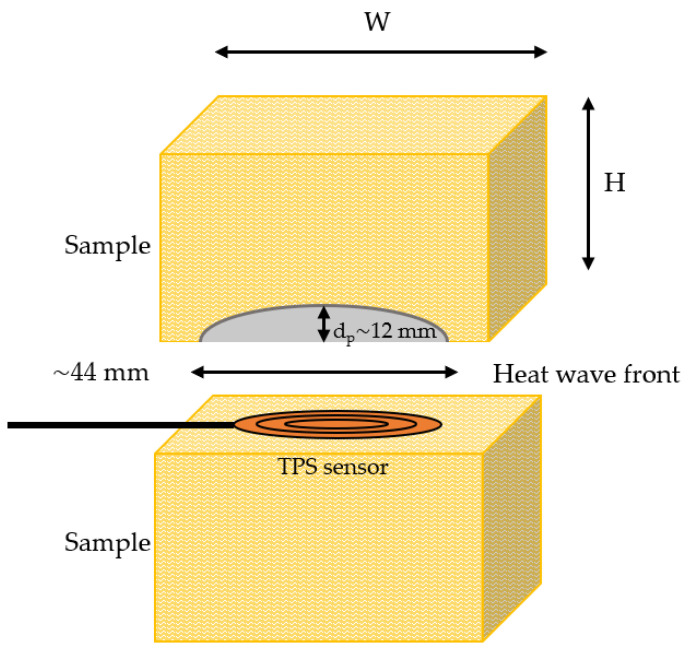
Principle sketch of the TPS measurement set-up.

However, when exposed to temperatures above 700 °C, the thermal insulation starts sintering, and changes considerably, especially at temperatures above 1100 °C. In the present study, thermal conductivity of heat-treated thermal insulation was recorded using the Transient Plane Source (TPS) method [31,32]. The TPS measurements for the heat-treated test specimens were performed using the Hot Disk Standard, double-sided according to [32] up to 700 °C, which is the temperature limit of this method. The pre-heated samples were cut in half and the TPS sensor was placed between the two sample halves, as shown in Figure 5.

It should be noted that new samples were made for the thermal conductivity measurements, i.e., without a thermocouple penetrating the insulation, as described in Section 2.2. Each recording reported in the present study is an average of three consecutive measurements with a relaxation time of 60 min between each measurement. The thermal conductivity results at temperatures up to 700 °C were further extrapolated up to the respective heat treatment temperature.

### 2.4. Density of Heat Treated Test Specimens

Based on the measured height and width after heat treatment, as described in Section 2.2, the volume of each test specimen was estimated. The mass of the test specimens was also measured after heat treatment. Based on the volume and the mass, the room temperature density as a function of heat treatment temperature was established.

### 2.5. Specific Heat

The specific heat of the thermal insulation as a function of temperature was calculated based on the given composition of the inorganic salts supplied by the manufacturer and presented in Appendix A. The data and equations used for calculating the specific heat for each inorganic salt, and for the final mixture, are presented in Appendix C.

Volumetric heat capacity, ρ∙*C*_p_ (J/m^2^K), may also be calculated from the TPS measurements based on the recorded thermal conductivity *k* (W/mK) and the thermal diffusivity *a* (m^2^/s), i.e., by ρ∙*C*_p_ = *k*/*a*.

## 3. Results

### 3.1. Dimensional Changes

**Figure 6:**
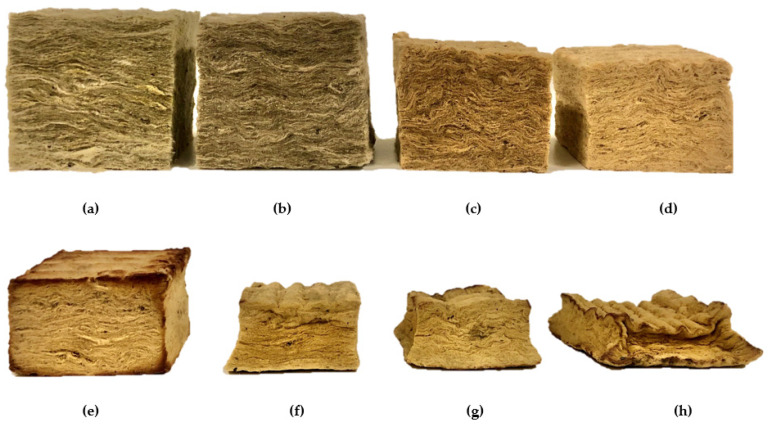
Thermal insulation after heating in muffle furnace to (**a**) virgin sample, (**b**) 700 °C, (**c**) 800 °C, (**d**) 1000 °C, (**e**) 1140 °C, (**f**) 1180 °C, (**g**) 1190 °C and (**h**) 1200 °C.

A significant shrinkage took place, especially at temperatures above 1100 °C, hence the temperature range above 1100 °C was of most interest. It was therefore decided to have a “finer mesh” when approaching 1200 °C, i.e., shorter temperature intervals between the heat treatment temperatures. Virgin thermal insulation and test specimens heat treated to 700 °C, 800 °C, 1000 °C, 1140 °C, 1180 °C, 1190 °C and 1200 °C are shown in Figure 6. It is clearly seen in Figure 6 that the “breaking point” of the insulation is around 1200 °C. It should be noted that the test specimen heat treated to 1200 °C, i.e., sample h, had an original size of 75 mm × 75 mm × 50 mm prior to heat treatment.

**Figure 7:**
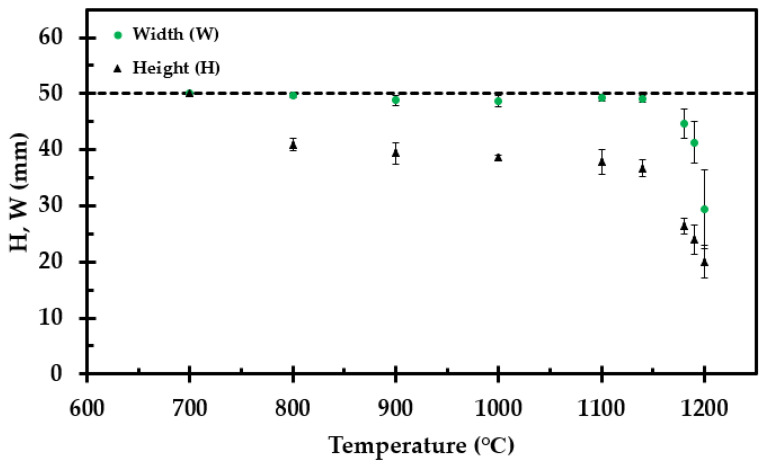
Height (H) and width (W) of the test specimen after heat treatment. The values represent an average of three measurements at each vertical side.

**Figure 8:**
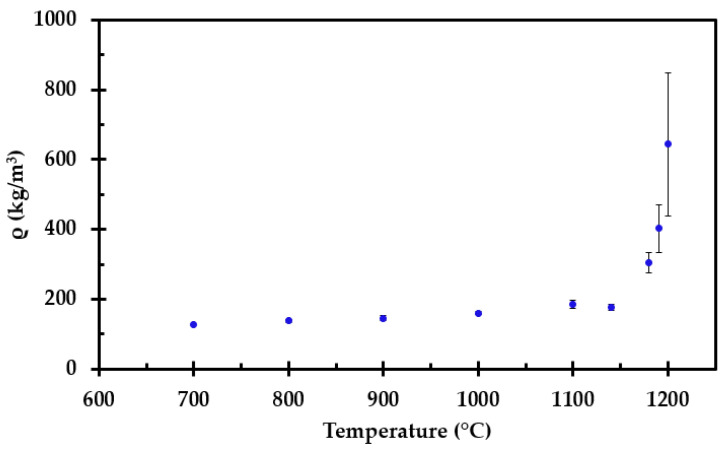
Calculated density at room temperature as a function of heat treatment temperature.

The measured height and width after heat treatment of each sample is presented in Figure 7 as a function of heat treatment temperature. For the sample heat treated to 1200 °C, the width and height in Figure 7 are normalized to a 50 mm virgin cube. The calculated density at room temperature is presented in Figure 8.

**Figure 9:**
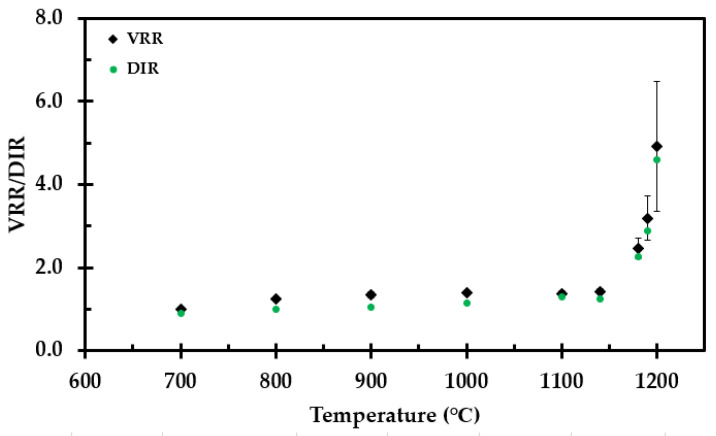
The volume reduction ratio (VRR) and density increase ratio (DIR) as a function the heat treatment temperature.

The corresponding volume reduction ratio (VRR) and density increase ratio (DIR) relative to the virgin test specimens are presented in Figure 9.

### 3.2. Thermal Conductivity Measurements

**Figure 10:**
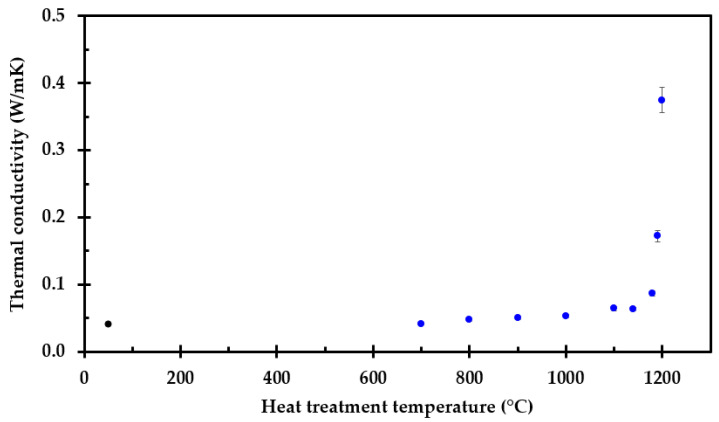
Ambient temperature thermal conductivity of heat-treated industrial thermal insulation measured by the TPS-method.

While the test specimens heat treated to temperatures of up to 1190 °C could be cut and measured using the TPS method, the 50 mm × 50 mm × 50 mm (height) thermal insulation test specimens heat treated at 1200 °C did shrink too much. It was therefore decided to make this test specimen from an original sample of 75 mm × 75 mm × 50 mm (height). The ambient temperature thermal conductivity of heat-treated thermal insulation is presented in Figure 10.

**Figure 11:**
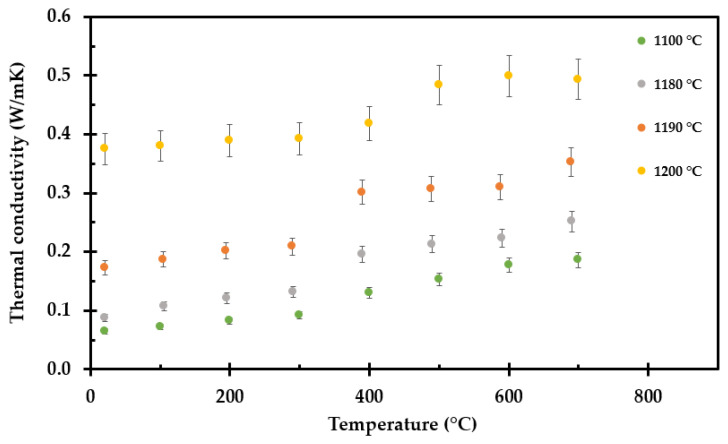
Results from TPS measurement of selected pre heated insulation samples, to 1100 °C, 1180 °C, 1190 °C and 1200 °C.

The thermal conductivity of all the test specimens, i.e., after heat exposure to 700 °C, 800 °C, 900 °C, 1000 °C, 1100 °C, 1140 °C, 1180 °C, 1190 °C and 1200 °C, was recorded by the TPS method from room temperature to 700 °C at each 100 °C interval. A selection of the thermal conductivity measurement results are shown in Figure 11, while all the measurement results are presented in Appendix B. The results represent an average of three measurements at each temperature, with 60 min relaxation time between consecutive measurements at the same temperature. The accuracy of the TPS method is ±2% to 5% at ambient temperatures and ±5% to 7% at elevated temperatures [32]. The thermal conductivity of the test specimens preheated to 800 °C and above seems to comply fairly well with a linear increase with increasing temperature.

**Figure 12:**
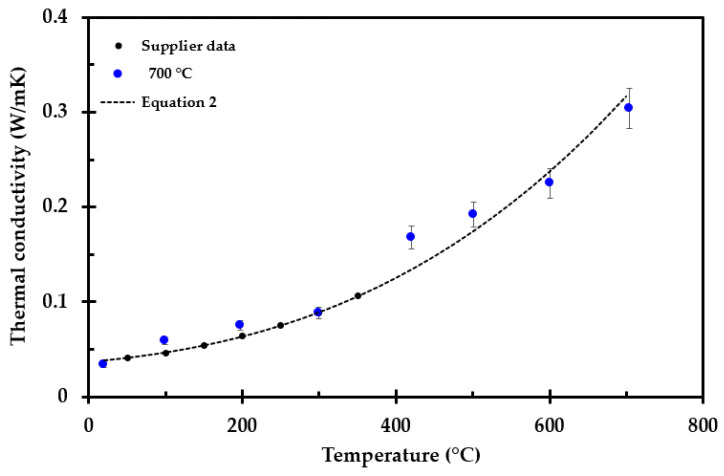
Thermal conductivity as a function of temperature for the test specimen preheated to 700 °C, supplier data available up to 350 °C and Equation (2) (---).

**Figure 13:**
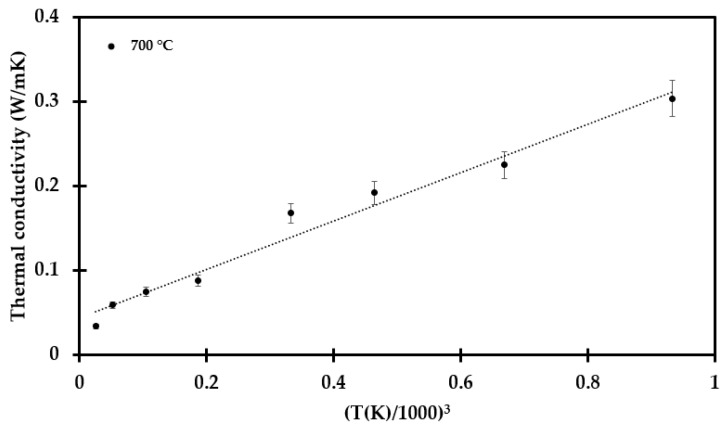
Thermal conductivity as a function of temperature to the third power for the test specimen heat treated to 700 °C. The linear function is presented in Appendix B, Equation (A1).

The only exception was the test specimen heat treated to 700 °C, as presented in Figure 12. It should be noted that the maximum operating temperature of the thermal insulation is 700 °C. Thus, upon heating to this temperature, there is little change in the thermal insulation, except for the loss of the dust binder and Bakelite materials. This may explain the results presented in Figure 6 for the test specimen treated at 700 °C, where the pore radiation may still dominate, i.e., the thermal conductivity versus temperature still follows Equation (2) quite well, as shown in Figure 12. The measured thermal conductivity as a function of absolute temperature to the third power is shown in Figure 13.

**Figure 14:**
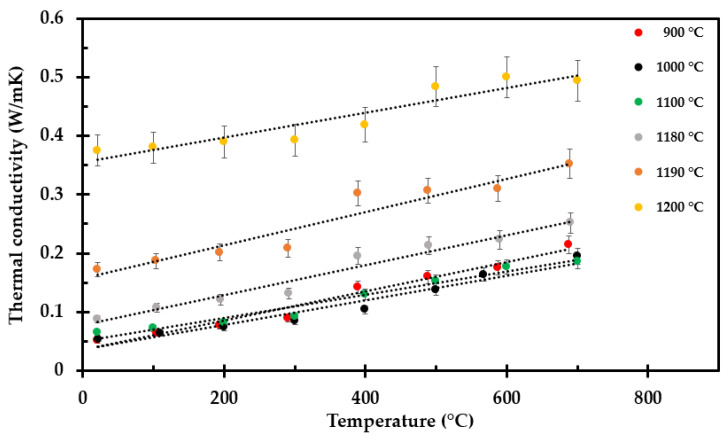
Thermal conductivity as a function of temperature for selected samples preheated to 900 °C, 1000 °C, 1100 °C, 1180 °C, 1190 °C and 1200 °C. Linear trend lines from Appendix B.

The samples preheated to 800 °C and above showed a more linear trend in thermal conductivity versus temperature, as seen in Figure 14. They also exhibit quite similar slopes (see Equations (A2)–(A8) for all the tested samples in Appendix B).

**Figure 15:**
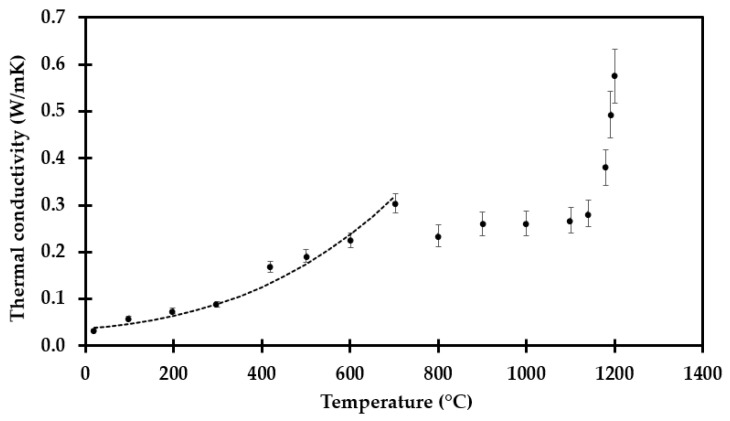
Thermal conductivity as a function of temperature, extrapolated to the respective heat treatment temperatures.

These results have been extrapolated to the respective heat treatment temperatures to gain an estimate of the true thermal conductivity at that temperature, which was above the temperature limit of the TPS method. The final results of this extrapolation are presented in Figure 15, which then represents a best estimate of the true thermal conductivity at that temperature.

At temperatures below 700 °C, i.e., Equation (2), the recorded thermal conductivity is highly dependent on the pore radiation, i.e., dependent on the absolute temperature to the third power. For the next 100 °C interval, sintering closes pores and results, counterintuitively, in lower thermal conductivity.

At temperatures above 800 °C, the increased level of sintering results in an increase in the recorded thermal conductivity, which increases very much between 1180 °C and 1200 °C. However, in future numerical modeling the shrinkage must also be taken into consideration, as it either influences the size of the grid studied or the effective thermal conductivity in a constant grid system.

The Fourier law of heat conduction is given by:

*q*_x_ = *k*∙Δ*T*/Δ*x* (W/mK), (3)

where Δ*x* (m) is along the path of heat conduction, i.e., during fire testing as shown in Figure 1, through the thickness of the thermal insulation mat. The results presented in Figure 13 are representative estimates for the thermal conductivity at these respective temperatures.

A simple way to correct for the shrinkage would be to still use the original thickness dimension of the thermal insulation in the modeling and adapt an apparent thermal conductivity, *k*_app_ (W/mK), correcting for the shrinkage, i.e.,:

*k*_app,x_ = *k*∙(*H*_o_/*H*_(T)_) (W/mK), (4)

where *H*_o_ (m) is the virgin thermal insulation mat thickness and *H*_(T)_ (m) is the thickness after heat treatment to temperature *T* (K), as presented in Figure 7. When correcting the thermal conductivity by the shrinkage factor, *H*_o_/*H*_(T)_, the numerical domain size may be considered constant.

**Figure 16:**
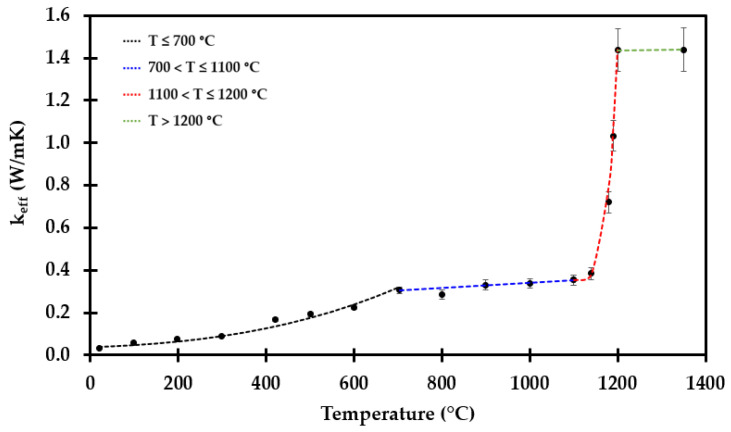
Apparent thermal conductivity as a function of temperature, i.e., adjusted for thermal insulation shrinkage, up to 1200 °C, represented by Equations (5)–(8).

The resulting apparent thermal conductivity, *k*_app_, at the respective heat treatment temperatures are presented in Figure 16.

It is clearly seen that this is a very complicated function of temperature, with empirical fits given in Equation (5) to Equation (8):

For T ≤ 700 °C:

*k*_eff, T ≤ 700 °C_ = 0.034 + 0.311⋅10^−9^⋅*T*^3^    (W/mK), (5)

for 700 °C < T ≤ 1100 °C:

*k*_eff, 700 °C < T ≤ 1100 °C_ = 0.216 + 1.254⋅10^3^⋅*T*   (W/mK) (6)

for 1100 °C < T ≤ 1200 °C:

*k*_eff, 1100 < T ≤ 1200 °C_ = 0.3537 + 1.084⋅10^−8^⋅(*T*−1100)^4^  (W/mK) (7)

and for T > 1200 °C:

*k*_eff, T > 1200 °C_ = 1.333 + 1.422⋅10^−5^⋅*T*  (W/mK), (8)

Previous thermal gravitational analysis (TGA)/differential thermal analysis (DTA), and differential scanning calorimetry (DSC) [20,22] revealed that critical phase changes take place close to a temperature of 1200 °C. It may then be assumed that only minor changes in inorganic salt concentrations may alter the thermal insulation properties considerably when approaching 1200 °C. The results obtained for heat treatment at 1200 °C may therefore be taken as an indication rather than as a robust estimate. This must be taken into consideration in future numerical modeling.

### 3.3. Volumetric Heat Capacity

**Figure 17:**
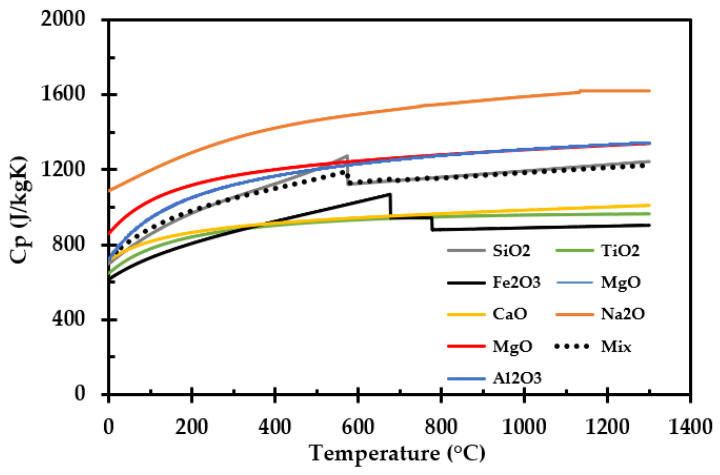
Calculated specific heat capacity as a function of temperature. Based on literature data presented in Appendix C.

The calculated specific heat as a function of temperature of each involved inorganic salt is presented in Figure 17. “Mix” represents the calculated specific heat of the thermal insulation inorganic salt mixture, as given in Appendix C.

**Figure 18:**
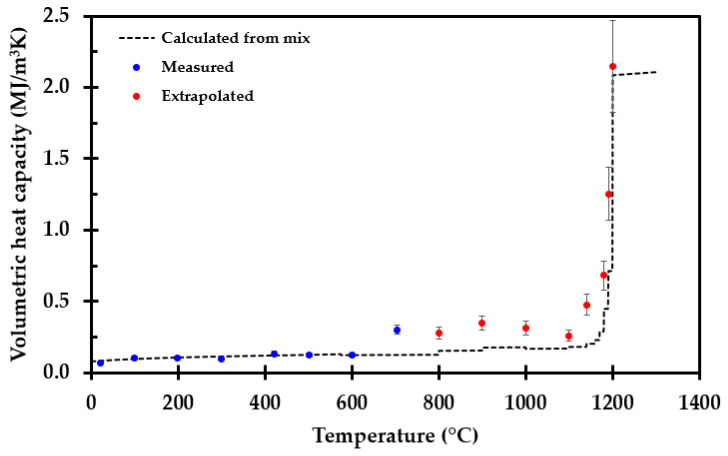
Measured and extrapolated volumetric heat capacity compared to the calculated volumetric heat capacity of the thermal insulation (---).

The specific heat may also be calculated from the TPS measurements. The strength of that measurement method is, however, the thermal conductivity, while estimates of the thermal diffusivity are generally less accurate. The extrapolated data from the TPS measurements compared to the calculated volumetric heat from the composition are shown in Figure 18. Due to the uncertainties in the TPS method, estimating specific heat from the chemical composition is believed to give a more accurate specific heat compared to the less reliable TPS data, which additionally represents extrapolated values.

### 3.4. Crack Formation

The shrinkage results in gaps in the insulation mat, as shown in Figure 2. During high temperature fire testing, these gaps may represent heat radiation shortcuts for the heat transfer from the heat-exposed cladding towards still-intact thermal insulation, and possibly to the perforated plate and the steel object to be protected. For future modelling, the heat radiation through these gaps also has to be modelled.

The cracking caused by the insulation shrinkage is temperature-dependent. As the temperature increases during fire testing, more cracking will occur deeper into the thermal insulation.

The shrinkage of the samples, presented in Figure 7, was measured in both directions, i.e., along the length and width of the thermal insulation mat of the originally 50 mm by 50 mm (long and wide) test specimens. The shrinkage in height (z-direction) is accounted for in the apparent thermal conductivity, while the shrinkage in the x- and y-direction may represent the cracking.

A simple approach to model the possible open area fraction, *A*_f_, would be to estimate it via the recorded test specimen length S_1_ (m), and width, S_2_ (m), of the thermal insulation mat, i.e.,

(9) $$A_{f}=1-\frac{S_{1}\;\cdot \;\;S_{2}}{50\;mm\;\cdot \;50\;mm}.$$

**Figure 19:**
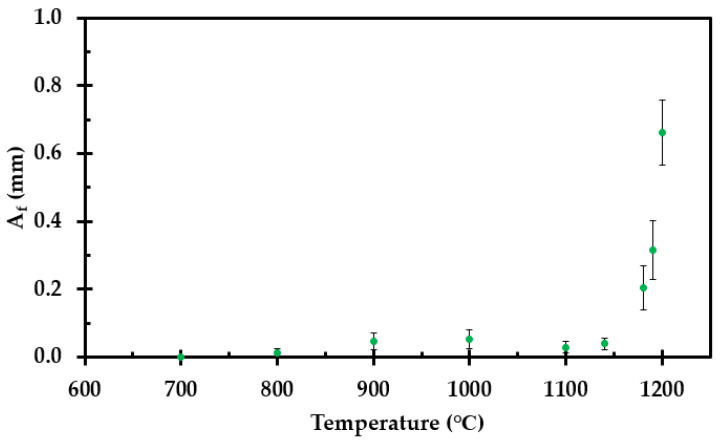
*A*_f_ as a function of temperature for the samples pre-heated to temperatures in the range 700 °C to 1200 °C.

The open area fraction as a function of heat treatment temperature is presented in Figure 19. Only a minor change in the open area would be expected at temperatures below, e.g., 1100 °C. Above this temperature, especially from, e.g., 1150 °C to 1200 °C, there is a significant increase in the open area fraction.

For possible future modeling, it should be noted that the cracks may not necessarily develop homogeneously through the heat-exposed thermal insulation. Seen along the path of heat radiation from the heat-exposed cladding towards the potentially heat-exposed pipes or equipment, the cracks may exhibit some tortuosity, i.e., where unevenly cracked parts block the direct heat radiation, partly resembling radiation shields. A way to model this needs to be developed, or a general tortuosity correction reducing the effective open area fraction may be applied. Introducing random locations of open fraction in each layer Δ*x* and Monte Carlo simulations to model the probability of different outcomes may also be a possibility.

### 3.5. Internal Temperature Development during Heat Treatment

**Figure 20:**
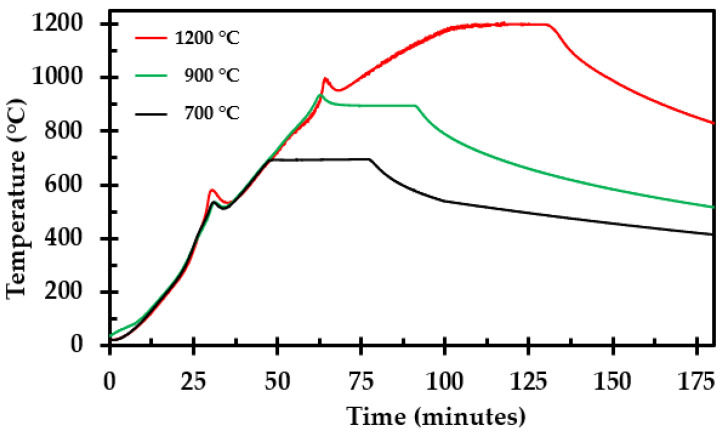
Internal temperature recorded during heat treatment to selected holding temperatures, i.e., 700 °C, 900 °C and 1200 °C.

The measured temperature in the center of the thermal insulation test specimen during heat treatment in the muffle furnace is presented in Figure 20. Two exothermic reactions were observed, presented as two temperature peaks during the heating, as also observed in the previous studies [20,22]. The first peak started around 300 °C and the second peak around 870 °C. As stated in the previous research [20,22], the first reaction may possibly be explained by the combustion of dust binder and Bakelite, while the second peak may be explained by an expected glass transition of the involved materials at temperatures in the range of 850 °C to 900 °C.

From the previously performed high-intensity fire tests (Figure 1), it was observed that the thermal insulation upon fire exposure released vapors that burned on the outside of the cladding, i.e., where oxygen was available for the combustion to take place. This indicates that during fire testing, and possibly also real fire exposure, there is limited air access into the thermal insulation being completely covered by stainless steel cladding. Whether this process, within the thermal insulation, represents a net heat gain or a heat drain is uncertain, while the glass transition represents a minor heat gain. It is, however, assumed that the net effects of binder pyrolysis and glass transition are small compared to the heat transfer taking place in the system. For a well-closed system, the enthalpy required for pyrolysis may indeed be partly, or fully, canceled out by the negative glass transition enthalpy.

## 4. Discussion

The aim of the present study was to investigate the industrial thermal insulation used at O&G sites in Norway and produce experimental data necessary for future numerical modeling of thermal insulation performance in fire exposure situations. The thermal conductivity and shrinkage experienced at elevated temperatures varies considerably with temperature, and ways to assess these have been tested. Previous studies have shown that thermal insulation alone may serve as a passive fire protection in cases where the steel represents a significant heat sink [19,20]. When the thermal insulation is exposed to temperatures above 700 °C, the insulation starts to lose height (mat thickness). When exposed to higher temperatures, i.e., from 1100 °C and above, the thermal insulation also starts to shrink in width and length (getting reduced base area). The VRR is, however, minor for temperatures below 1100 °C and significantly less than observed for, e.g., perlite compaction optimized by K_2_CO_3_ flux at 700 °C [33]. When exposed to higher temperatures, i.e., from 1100 °C and above, the thermal isolation also starts to shrink in width, i.e., along the fibers. The aim of the present study was to investigate the thermal conductivity of the thermal insulation and shrinkage experienced at elevated temperatures and suggest possibilities for future modeling of the heat transfer through the thermal insulation during fire testing. However, concepts for analyzing gap formation also have to be included in a prospective model.

The industrial thermal insulation studied is a material with concentrations of inorganic salts varying within given limits (Table A3), i.e., the composition of the insulation may determine the eutectic point of the insulation, and hence this may vary between different production batches. This may be seen from the muffle furnace tests performed in this study, compared with the muffle furnace tests performed in the previous study [17], considering samples heat treated to 1200 °C. There is a clear difference between the two samples, both visually and as demonstrated by the measured density. The thermal insulation changed from a soft, porous consistency to a stone like material after exposure to 1200 °C. This is more obvious in [22], where it shows total breakdown/melting of the insulation. In the present study, the thermal insulation had still shrunk considerably in all directions; however, clear signs that melting had taken place were not observed. Considering flame, or cladding, temperatures of 1200 °C, an, e.g., ±20 °C variation in the eutectic temperature could have a large influence on the performance of the thermal insulation when exposed to a cladding temperature of 1200 °C. The shrinkage of the insulation test specimen is not proportional—the insulation may shrink more in one direction than another, especially at temperatures above 1100 °C, as shown in Figure 6. Hence, the measurement of the remaining test specimen volume is a “best estimate” for a random thermal insulation batch.

It was demonstrated that the thermal conductivity is dominated by heat radiation within the pores up to 700 °C, i.e., the thermal conductivity increased with absolute temperature to the third power. The reported ambient thermal conductivity is slightly higher in the present study compared to the previously reported ambient thermal conductivity in [22]. This may be explained by differences between different thermal insulation batches, e.g., chemically or by different weaving of the thermal insulation fibers.

With further temperature increase, the thermal conductivity decreased due to sintering and pore size reduction at 800 °C. Above 900 °C, i.e., above the glass transition temperature, the thermal conductivity increased with temperature as expected for an amorphous material. Additionally, further compacted by sintering, and finally by partly melting as it approached an assumed eutectic temperature close to 1200 °C, the thermal conductivity increased with temperature.

It may seem quite strange that the thermal conductivity increases up to 700 °C, for it then to drop significantly at 800 °C, and above this temperature it starts increasing again with increasing temperature. This may, however, be explained by the fact that when preheating the insulation to 700 °C, there are limited dimensional changes in the inorganic insulation components, i.e., the pore radiation is still dominating. The sample preheated to 900 °C and above shrank in height and was more compact. These samples are amorphous, explaining the drop in the conductivity, evidenced by the conductivity following a more linear trend with increasing temperature [34]. From 700 °C to 900 °C, there seems to be a drop in the thermal conductivity. This may be explained be the sintering process that starts just above 700 °C, before the transition to a more amorphous material at around 900 °C. Above 1100 °C, the breakdown of the thermal insulation accelerates, also affecting the thermal conductivity. The concept of apparent thermal conductivity was introduced to compensate for the observed shrinkage along the insulation mat thickness. This apparent thermal conductivity may enable/enhance future numerical modeling.

Extrapolating the thermal conductivity results to heat treatment temperatures beyond 700 °C increases the ±7% uncertainty in the recorded thermal conductivity. Up to 1100 °C, it may seem reasonable to extrapolate the trend, as there is little change in the insulation. However, extrapolation up to temperatures near the eutectic point, where there is a significant change in the thermal insulation properties, there is a much higher degree of uncertainty in the results. Hence, a ±10% uncertainty is considered as an uncertainty estimate. As the chemical compositions of the different thermal insulation batches may vary some, especially the recorded and extrapolated results at heat treatment temperatures close to 1200 °C may be different from one batch to another batch. Thus, the differences in thermal conductivity at these temperatures may deviate even more for a random thermal insulation batch. Studying this variation was, however, not within the scope of the present study.

As for the upper temperature region, this may also apply for the lowest region of the testing, regarding the measurements around 700 °C. Small differences between insulation batches may influence the sintering process and at which temperature sintering starts. Hence, the results from the TPS measurements may represent a larger uncertainty, depending on the insulation batch.

The estimated value for the apparent thermal conductivity was, for the sake of simplicity, presented as three equations. Considering Equation (6) for the 700 °C to 1000 °C region, the equations fit quite well for all the involved measurements points, except for the extrapolated value at 900 °C, where it misses by approximately 10%. However, it does give a conservative estimate. The same deviation can be seen in one of the measurement points in Equation (7), regarding the region from 1100 °C to 1200 °C. However, the equations give a good estimate of the overall thermal conductivity.

At temperatures above 1100 °C, heat radiation through cracks will likely dominate the heat transfer through the degraded thermal insulation. The shrinkage of the insulation, that results in cracks, may be more difficult, but highly necessary, to model properly. The cracks may be quite random and may not necessarily penetrate the insulation, i.e., the cracks may exhibit some tortuosity along the main direction of heat radiation from the fire-exposed cladding to the object to be protected. In the present study, an estimate of the open fraction has been made based upon the shrinkage in the two principal directions, i.e., along the thermal insulation mat width and length. This was done as a function of temperature based upon the results from the muffle furnace tests. However, a way to model the cracks and heat transfer through the cracks must be developed. Alternatively, a general tortuosity correction reducing the effective open area could potentially be applied. Introducing random locations of the estimated open fraction in each layer of thickness Δ*x* and using Monte Carlo simulations to model the probability of different outcomes may also be considered.

The specific heat was calculated from the chemical composition of the thermal insulation and with knowledge of the density, the corresponding volumetric heat capacity was obtained. The volumetric heat capacity was also estimated based on the TPS measurements, i.e., based on the recorded thermal conductivity and thermal diffusivity [32]. The TPS method is not as accurate for measuring thermal diffusivity as for measuring thermal conductivity. Thus, there is a higher degree of uncertainty in the volumetric heat capacity obtained by the TPS method. When performing TPS measurements at 700 °C for the sample previously heat treated at 700 °C, further sintering of the insulation may have resulted in poor contact between the thermal insulation and the sensor, influencing the measurement results. This may be an explanation for the differences between the two methods for assessing volumetric heat capacity, as seen in Figure 18. Hence, calculating specific heat from literature data is recommended as the most reliable method.

During the muffle furnace heat treatment, there was air access to the interior of the test specimens. Thus, dust binder and Bakelite pyrolysis, and subsequent oxidation, clearly resulted in a net heating of the test specimens. However, during fire testing, as shown in Figure 1, it is more unclear as to whether there is a net gain or drain of heat from the much more sealed thermal insulation. During high-intensity fire tests (Figure 1), it was observed that upon fire exposure, the thermal insulation released vapors that ignited, and burned, on the outside of the cladding, i.e., in contact with air. This indicates that during fire testing, and possibly also real fire exposure, there is very limited air access into the thermal insulation which is completely covered by stainless steel cladding. The glass transition represents a minor heat gain, which may partly or fully cancel out the heat required for binder material and Bakelite pyrolysis. Further TGA/DSC tests may be necessary to answer this question.

There are some differences in the results obtained for test specimens heat treated to 1200 °C compared to similar tests [32]. However, due to the different compositions and non-homogeneities in the thermal insulation, the breakdown temperature of the thermal insulation will not be absolute. It is therefore recommended to test several different insulation mats, from different production batches, to check whether there are large differences between the batches or not. In the first place, this may be done by, e.g., muffle furnace heat treatment as in the present study and/or TGA/DSC, to verify any differences in melting temperature between the batches. It is also recommended to study the influence of the holding time on the thermal conductivity and geometric dimensions of the heat-treated test specimens. It should be mentioned that thermal insulation and fire protection may result in corrosion. Hence, fire protection should therefore only be used where strictly required [9].

When pipes and equipment are already thermally insulated with the product studied in the present work, adding a thin layer of passive fire protection could for an extended period prevent the thermal insulation from reaching temperatures above 1100 °C. This could prevent significant dimensional changes, cracks, etc. in the thermal insulation under fire exposure. Testing this in the future would be very beneficial. Such studies may reveal that the thermal insulation under such circumstances may survive quite extended periods of fire exposure.

## 5. Conclusions

By heat treating thermal insulation test specimens and recording the thermal conductivity up to 700 °C, and extrapolating to heat treatment temperatures above this value, it was possible to obtain an estimate of the thermal conductivity during fire exposure. This is expressed by empirical equations. However, as the temperature passes 1100 °C, the data will become less reliable, especially when approaching 1200 °C, i.e., where the thermal insulation disintegrates. For future numerical modeling of the heat flow through the thermal insulation to a steel member, shrinkage must also be considered, as this results in an open area fraction and more direct heat transfer. The promising behavior up to 1100 °C is, however, great news, as it may be possible to apply a layer of thermally robust fire insulation preventing the thermal insulation to reach temperatures above 1100 °C. This would extend the fire resistance of the thermal insulation considerably and could reduce the demand for blowdown and flare system capacity in aging O&G industry sites. The data obtained in the present study may allow for future numerical modeling of such fire exposure scenarios.

## Data Availability

Data available on request.

## Appendix A

The technical data for the thermal insulation are given in Table A1 and Table A2. The chemical composition of the thermal insulation is given in Table A3.

**Table A1:** Technical data for the Rockwool Pipe section mat thermal insulation [21].

**Name**	**Description**	
Material	Stone wool	
Operating range	−40 to 700 °C	
**Name**	**Performance**	**Norms**
Maximum service temperature	700 °C	EN 14706
Reaction to fire	Euroclass A1	EN 13501-1
Nominal density	140 kg/m^3^	EN 1602
Water absorption	≤1 kg/m^2^ ≤20 kg/m^3^	EN 1609
		BP 172
Water vapor diffusion resistance	Sd > 200 m	EN 12086
Air flow resistivity	>60 kPa·s/m^2^	
Designation code	MW EN 14303-T4-ST(+)700-WS1-MV2	EN 14303

**Table A2:** Thermal conductivity of the thermal insulation studied (Rockwool ProRox PSM 971. 50 mm) [21].

Temperature (°C)	Thermal Conductivity (W/m∙K)
50	0.041
100	0.046
150	0.054
200	0.064
250	0.075
300	0.088
350	0.106

**Table A3:** Data for the thermal insulation studied (Rockwool ProRox PSM 971. 50 mm) [35].

Name	Product	Percentage
Dust binder ^1^	Oil product	<0.5%
Binder ^1^	(C_6_H_6_O·CH_2_O)_N_	2.5% (±0.4%)
Bulk oxide	SiO_2_	40.6–44.6%
Bulk oxide	Al_2_O_3_	17.4–20.4%
Bulk oxide	MgO + CaO	23.9–27.9%
Bulk oxide	Fe_2_O_3_	5.5–8.5%
Bulk oxide	Na_2_O + K_2_O	1.3–4.3%
Bulk oxide	TiO_2_	0.6–2.6%
Bulk oxide	P_2_O_5_	Max. 1.2%

^1^ The binder calorific value is 27 MJ/kg according to ISO 1716.

## Appendix B

Equations for the measured thermal conductivity presented in Figure 12 and Figure 13.

(A1) $$y_{700\;{}^{\circ}C,\;{\left(\frac{T\left(K\right)}{1000}\right)}^{3}}=0.2873x+0.04369,\;R^{2}=0.9742$$

(A2) $$y_{800\;{}^{\circ}C}=2.53\;\cdot 10^{-4}\cdot T_{\left(℃\right)}+0.0316,\;R^{2}=0.9731$$

(A3) $$y_{900\;{}^{\circ}C}=2.50\cdot 10^{-4}\cdot T_{\left(℃\right)}+0.0352,{\;R}^{2}=0.9722$$

(A4) $$y_{1000\;{}^{\circ}C}=2.11\cdot 10^{-4}\cdot T_{\left(℃\right)}+0.0356,{\;R}^{2}=0.9512$$

(A5) $$y_{1100\;{}^{\circ}C}=1.98\cdot 10^{-4}\cdot T_{\left(℃\right)}+0.0498,{\;R}^{2}=0.9665$$

(A6) $$y_{1140\;{}^{\circ}C}=1.97\cdot 10^{-4}\cdot T_{\left(℃\right)}+0.0573,\;R^{2}=0.9633$$

(A7) $$y_{1180\;{}^{\circ}C}=2.57\cdot 10^{-4}\cdot T_{\left(℃\right)}+0.0773,\;R^{2}=0.9639$$

(A8) $$y_{1190\;{}^{\circ}C}=2.83\cdot 10^{-4}\cdot T_{\left(℃\right)}+0.157,\;R^{2}=0.9227$$

(A9) $$y_{1200\;{}^{\circ}C}=2.11\cdot 10^{-4}\cdot T_{\left(℃\right)}+0.355,\;R^{2}=0.8851$$

## Appendix C

The heat capacity of the insulation mixture is calculated from Equation (A10), based on data given in Table A4.

(A10) $$C_{p}=A+B\cdot T+C\cdot T^{2}+D\cdot T^{3}+\frac{E}{T^{2}}$$

**Table A4:** Data for the components in the thermal insulation given in Table A3 [36,37,38,39,40,41,42,43].

**Percentage (%)**	42.54	19.33	1.64	7.16	13.24	13.24	1.43	1.43
**Product**	SiO_2_	Al_2_O3	TiO_2_	Fe_2_O_3_	MgO	CaO	Na_2_O	K_2_O
**Mw (g/mol)**	60.0843	101.9613	79.866	159.688	40.3044	56.077	61.9789	94.196
**Mw (kg/mol)**	0.0600843	0.1019613	0.079866	0.159688	0.0403044	0.056077	0.0619789	0.094196
**T (°C)**	**298–847**	**298–2327**	**298–2000**	**298–950**	**298–3105**	**298–3200**	**298–1023**	**298–700**
A	−6.076591	106.918	67.2983	93.43834	47.25995	49.95403	25.5754	245.0104
B	251.6755	36.6219	18.7094	108.3577	5.681621	4.887916	177.71	−567.0492
C	−324.7964	−13.9759	−11.579	−50.86447	−0.872665	−0.352056	−166.335	778.7219
D	168.5604	2.15799	2.449561	25.58683	0.1043	0.046187	57.6116	−346.2641
E	0.002548	−3.157761	−1.485471	−1.61133	−1.053955	−0.825097	0.338149	−4.653361
**T (°C)**	**847–1996**	**-**	**-**	**950–1050**	**-**	**-**	**1023–1243**	**700–2000**
A	58.7534			150.624			−125.773	72.55098
B	10.27925			0			302.074	41.39097
C	−0.131384			0			−140.642	−0.728497
D	0.02521			0			21.324	0.218564
E	0.025601			0			38.2831	0.066026
**T (°C)**	**-**	**-**	**-**	**1050–2500**	**-**	**-**	**1243–1405**	**-**
A				110.9362			2240.95	
B				32.04714			−3209.97	
C				−9.192333			1803.69	
D				0.901506			−359.073	
E				5.433677			−386.248	
